# Supplementary material for: A computer architecture based on disruptive information technologies for drug management in hospitals
Source: PeerJ Comput Sci. 2023 Jun 29;9:e1455. doi: 10.7717/peerj-cs.1455 (PMC10319265; doi:10.7717/peerj-cs.1455)
Supplement: Supplemental Information 1 [file peerj-cs-09-1455-s001.pdf]

QUALITATIVE EVALUATION

Expert's background:

Three academics, university assistant professors, with more than ten years working as teachers and researchers. Two of them (experts 1 and 2) from the Department of Nursing and one from the Department of Computer Science (experts 3).

Three practitioners, two belonging to the Department of Pharmacology of a hospital with extensive experience in drug management in hospitals (experts 4 and 5), and one belonging to a Computer Consultancy and Project Development company with experience in disruptive information technologies (expert 6).

Procedure:

- Explanation of the existing problems related to drug traceability in hospitals
- Description of the computer architecture proposed
- Experts opinions' collection

Questions:

1. Do you think that the computer architecture proposed can be useful for drug management in hospitals?
2. Do you think that the structure of the computer architecture is adequate?
3. Do you think that proposed architecture is complete, clear and the level of detail is appropriate?
4. How you found any mistake?
5. Would you suggest any change for improvement?
6. Which are the main strengths and weaknesses of the computer architecture, comparing it with the current computer architectures for drug management in hospitals?

Answers:

The following table shows a synthesis of the expert's answers

**Expert**	**Question 1**	**Question 2**	**Question 3**	**Question 4**	**Question 5**	**Question 6**
**1**	Yes	Yes	Yes	Yes	Yes	Yes
**2**	Yes	Yes	Yes	Yes	Yes	Yes
**3**	It is clear and the level of detail is adequate. However, some changes are needed	It is clear and the level of detail is adequate. However, some changes are needed	Yes	It is clear and the level of detail is adequate. However, some changes are needed	Yes	Yes
**4**	No	No	No	No	No	No
**5**	No	No	Yes<br>It is necessary to explain when to	Yes<br>It is necessary to consider	Yes<br>It is necessary to consider	No

			use Iot, RFid and QR codes	data privacy issues	data privacy issues	
**6**	The computer architecture covers the three phases of drug data management in hospitals and addresses existing problems related to drug traceability in hospitals	The computer architecture will allow data exploitation to improve decisions making	The proposal of a third generation Business Intelligence system to support decision making related to drugs in hospitals.<br>The proposal of a private Blockchain instead of a public Blockchain	The computer architecture will allow an adequate drug traceability in hospitals	The computer architecture will allow an adequate drug traceability in hospitals	The integration of smart contracts inside the Business Intelligence system.<br>The proposal of a private Blockchain instead of a public Blockchain
